# Radiomics model based on intratumoral and peritumoral features for predicting major pathological response in non-small cell lung cancer receiving neoadjuvant immunochemotherapy

**DOI:** 10.3389/fonc.2024.1348678

**Published:** 2024-03-20

**Authors:** Dingpin Huang, Chen Lin, Yangyang Jiang, Enhui Xin, Fangyi Xu, Yi Gan, Rui Xu, Fang Wang, Haiping Zhang, Kaihua Lou, Lei Shi, Hongjie Hu

**Affiliations:** ^1^ Department of Radiology, Sir Run Run Shaw Hospital, Zhejiang University School of Medicine, Hangzhou, Zhejiang, China; ^2^ Department of Radiology, The First Affiliated Hospital of Wenzhou Medical University, Wenzhou, Zhejiang, China; ^3^ Medical Imaging International Scientific and Technological Cooperation Base of Zhejiang Province, Sir Run Run Shaw Hospital, Zhejiang University School of Medicine, Hangzhou, Zhejiang, China; ^4^ Department of Radiology, Zhejiang Cancer Hospital, Hangzhou Institute of Medicine (HIM), Chinese Academy of Sciences, Hangzhou, Zhejiang, China; ^5^ Department of Research and Development, Shanghai United Imaging Intelligence Co., Ltd., Shanghai, China; ^6^ Department of Pathology, Sir Run Run Shaw Hospital, Zhejiang University School of Medicine, Hangzhou, Zhejiang, China; ^7^ DUT-RU International School of Information Science and Engineering, Dalian University of Technology, Dalian, Liaoning, China; ^8^ DUT-RU Co-Research Center of Advanced Information Computing Technology (ICT) for Active Life, Dalian University of Technology, Dalian, Liaoning, China

**Keywords:** lung neoplasms, machine learning, immunotherapy, neoadjuvant therapy, peritumor

## Abstract

**Objective:**

To establish a radiomics model based on intratumoral and peritumoral features extracted from pre-treatment CT to predict the major pathological response (MPR) in patients with non-small cell lung cancer (NSCLC) receiving neoadjuvant immunochemotherapy.

**Methods:**

A total of 148 NSCLC patients who underwent neoadjuvant immunochemotherapy from two centers (SRRSH and ZCH) were retrospectively included. The SRRSH dataset (n=105) was used as the training and internal validation cohort. Radiomics features of intratumoral (T) and peritumoral regions (P1 = 0-5mm, P2 = 5-10mm, and P3 = 10-15mm) were extracted from pre-treatment CT. Intra- and inter- class correlation coefficients and least absolute shrinkage and selection operator were used to feature selection. Four single ROI models mentioned above and a combined radiomics (CR: T+P1+P2+P3) model were established by using machine learning algorithms. Clinical factors were selected to construct the combined radiomics-clinical (CRC) model, which was validated in the external center ZCH (n=43). The performance of the models was assessed by DeLong test, calibration curve and decision curve analysis.

**Results:**

Histopathological type was the only independent clinical risk factor. The model CR with eight selected radiomics features demonstrated a good predictive performance in the internal validation (AUC=0.810) and significantly improved than the model T (AUC=0.810 vs 0.619, p<0.05). The model CRC yielded the best predictive capability (AUC=0.814) and obtained satisfactory performance in the independent external test set (AUC=0.768, 95% CI: 0.62-0.91).

**Conclusion:**

We established a CRC model that incorporates intratumoral and peritumoral features and histopathological type, providing an effective approach for selecting NSCLC patients suitable for neoadjuvant immunochemotherapy.

## 1 Introduction

Lung cancer has emerged as the leading cause of cancer-related deaths worldwide (1). Among them, non-small cell lung cancer (NSCLC) accounts for approximately 85% (2). The past decade of lung cancer treatment history has demonstrated that preoperative administration of antitumor drugs can reduce tumor size, leading to downstaging and creating favorable conditions for surgery (3). Additionally, research has indicated that neoadjuvant therapy can help eliminate micrometastases and reduce the risk of post-operative recurrence (4). With the advancement of lung cancer treatment drugs, immune checkpoint inhibitors have emerged as a novel and promising class of antitumor agents (5, 6). Studies have shown that the addition of nivolumab to neoadjuvant chemotherapy in lung cancer significantly improves pathological response in patients compared to the use of chemotherapy alone (7, 8).

However, only part of NSCLC patients can benefit from neoadjuvant immunochemotherapy (7). In many cases, the tumor did not shrink significantly following neoadjuvant therapy, and these drugs can have notable side effects such as leukopenia and immune-related pneumonitis (3, 9). Therefore, it is crucial to identify patients who will truly benefit from neoadjuvant immunochemotherapy before initiating treatment (10). In fact, assessing the efficacy of neoadjuvant therapy in lung cancer poses certain challenges, as studying the survival outcomes of patients after neoadjuvant treatment typically requires a long time follow-up (11). The International Association for the Study of Lung Cancer (IASLC) in 2021 suggested that the major pathological response (MPR) in postoperative specimens can be used as an evaluation criterion for neoadjuvant therapy (12). MPR was defined as the viable tumor is less than or equal to 10% in the tumor bed, which provided a convenient approach to assessing treatment effectiveness after neoadjuvant therapy.

Some clinical trials have explored the use of biomarkers such as PD-L1 expression and tumor mutational burden (TMB) to predict MPR. However, their predictive effectiveness remained controversial and the detection of PD-L1 and TMB is invasive. To date, there is no reliable biomarker available to predict MPR following neoadjuvant immunochemotherapy in NSCLC. Thus, there is an urgent need for a credible and non-invasive pre-treatment assessment method.

Radiomics aims to capture the heterogeneity within tumors non-invasively by extracting high-throughput features from images for analysis (13). Numerous studies have demonstrated that radiomics plays a valuable role in tumor diagnosis, treatment, and prognosis assessment (14–16). Research has already utilized pre-treatment CT tumor features to build radiomics model and predict pathological response following neoadjuvant chemoradiation for lung cancer, yielding promising results (17). In fact, the microenvironment surrounding the tumor can also influence the response to immunotherapy, such as the distribution of tumor-infiltrating lymphocytes (TILs) (18). Studies have shown that the distribution of TILs is associated with survival outcomes and treatment response in various diseases (19, 20). Therefore, it is also necessary to further investigate the impact of the specificity of the tumor microenvironment on the effectiveness of neoadjuvant immunochemotherapy.

In this study, we constructed models to predict MPR following neoadjuvant immunochemotherapy for non-small cell lung cancer by extracting radiomic features from both the intratumor and the peritumor regions on CT images. Furthermore, the optimal prediction model was validated in an independent external cohort.

## 2 Methods and materials

### 2.1 Study population

This study was granted ethical approval by the institutional review board of Sir Run Run Shaw Hospital (SRRSH) and Zhejiang Cancer Hospital (ZCH), which was performed in accordance with the ethical standards of the 1964 Declaration of Helsinki. Informed consent was waived due to the retrospective nature of this study.

**Figure 1:**
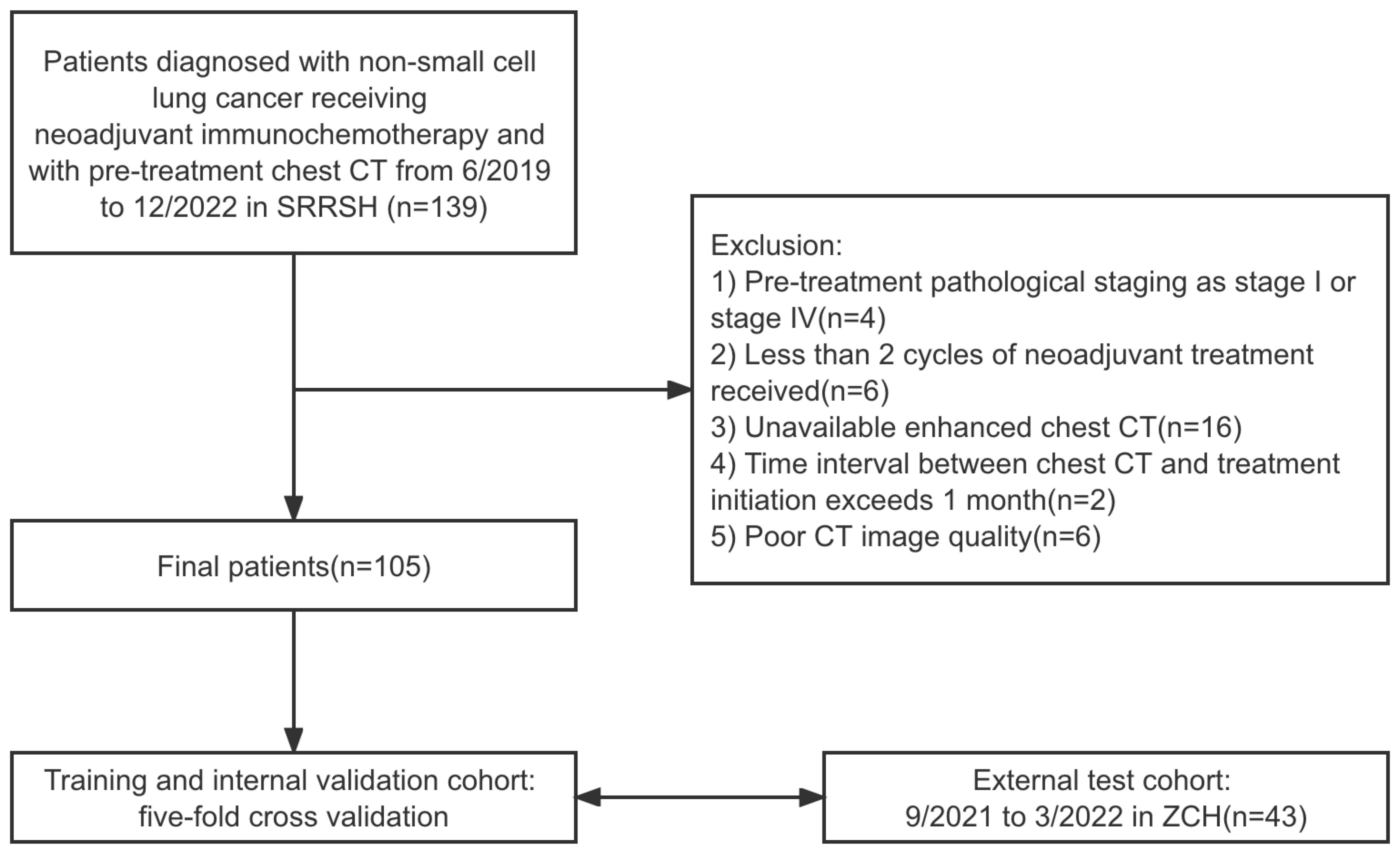
Patient selection and distribution flowchart.

This research retrospectively included patients diagnosed with non-small cell lung cancer (NSCLC) who underwent neoadjuvant immunochemotherapy between June 2019 and December 2022 at two centers (SRRSH and ZCH). The inclusion criteria were as follows: 1) pathologically confirmed NSCLC through endoscopic bronchoscopy or CT-guided needle puncture, 2) preoperative neoadjuvant immunochemotherapy was received, and 3) pre-treatment chest CT was performed. Patients were excluded if any of the following conditions were met: 1) pre-treatment staging as stage I or stage IV; 2) less than two cycles of neoadjuvant treatment received; 3) unavailable enhanced chest CT; 4) time interval between chest CT and treatment initiation exceeds one month; 5) poor CT image quality. Patients from SRRSH were used as the model training and internal validation set, while patients from ZCH were used as the independent external test set. The detailed process of patient inclusion and exclusion is shown in **Figure 1**.

### 2.2 Treatment method

All patients underwent standard preoperative evaluations and tumor staging procedures before determining treatment, including tumor biopsy (via bronchoscopy or CT-guided fine-needle puncture), chest CT, abdominal ultrasound, head MRI, and whole-body nuclear imaging. The thoracic surgeons assessed the tumor staging of the patients according to the 8th edition of the Lung Cancer TNM staging system, published by the Union for International Cancer Control (UICC), and determined the neoadjuvant treatment strategy. The standard neoadjuvant immunochemotherapy regimen comprises 2 to 4 cycles of immunotherapy in conjunction with platinum-based chemotherapy. Following the completion of neoadjuvant treatment, comprehensive tumor resection is undertaken by the thoracic surgeons.

### 2.3 Pathological evaluation

According to the multidisciplinary recommendations from the IASLC regarding pathological assessment of lung cancer excision specimens after neoadjuvant therapy (12), pathologists are responsible for evaluating the pathological responses of surgical specimens. All specimens were re-evaluated by an experienced senior pathologist (Y. Gan) who has more than 10 years of experience in accordance with IASLC. If the initial pathology report is different from Dr. Gan’s, Dr. Gan’s opinion shall prevail. MPR is defined as the percentage of viable tumor cells in the tumor bed being less or equal to 10%. Non-MPR is defined as the percentage of residual tumor cells in the tumor bed more than 10%.

### 2.4 Image acquisition

The CT scanning parameters in the two centers are shown in **Table 1**. The contrast-enhanced scanning technique involved intravenous injection of nonionic contrast material (Ultravist 300 or Ultravist 370, Bayer; or ioversol 320, Hengrui) at a rate of 2.2 to 3 ml/s, based on a dosage of 1.2 ml/kg body weight. Bolus tracking technique was employed, with the arterial phase scan initiated 8 seconds after the descending aortic CT density reached 100 HU. All CT scans were retrieved from the picture archiving and communication system (PACS) for further feature extraction.

**Table 1:** Scanning parameters and CT specifications in both hospitals.

	Sir Run Run Shaw Hospital					Zhejiang Cancer Hospital		
Brand	Siemens	Siemens	Siemens	GE	GE	Siemens	GE	Philips
Machine type	SOMATOM Definition Flash	SOMATOM Force	SOMATOM go. Top	Lightspeed VCT	Optima CT620	SOMATOM Definition Flash	Optima CT680	Ingenuity CT
Tube voltage (KV)	120	100/120	120	120	120	120	120	120
Tube current (mAs)	smart	smart	smart	smart	smart	smart	smart	smart
Rotation time (s)	0.5	0.5	0.5	0.4、0.5	0.5	0.5	0.5	0.5
Image matrix	512×512	512×512	512×512	512×512	512×512	512×512	512×512	512×512
Field of view (mm)	350	350	350	350	350	350	350	350
Reconstruction slice thickness and spacing	2mm/2mm	2mm/2mm	2mm/2mm	1.25mm/1.25mm	2mm/2mm	2mm/2mm	5mm/5mm	1.25mm/1.25mm
Reconstruction algorithm	B41f	B41f	B41f	Standard resolution	Standard resolution	B31f	Standard resolution	Standard resolution

### 2.5 Radiomics procedures

The workflow of radiomics analysis consisted of five steps: region of interest (ROI) segmentation, radiomics features extraction and selection, model construction and evaluation. Radiomics analysis was performed with uAI Research Portal (United Imaging Intelligence, China) (21), which is a clinical research platform implemented by Python programming language (version 3.7.3), and widely used package PyRadiomics (https://pyradiomics.readthedocs.io/en/latest/index.html).

**Figure 2:**
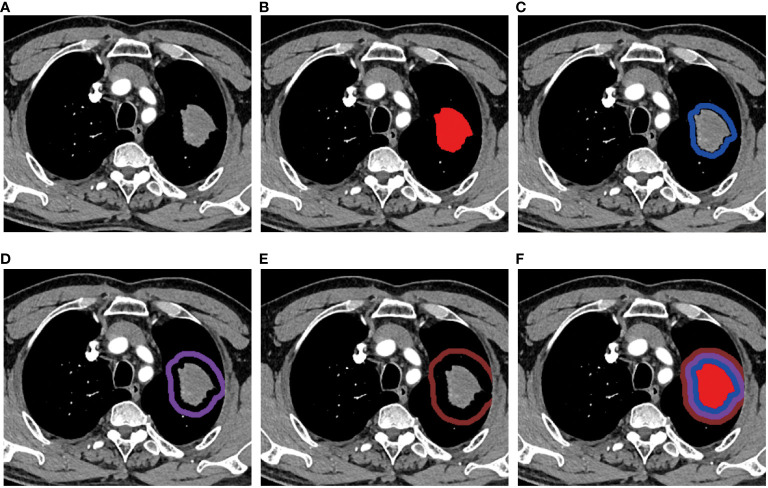
Region of interest (ROI) segmentation. **(A)** A mass showed in the upper lobe of the left lung. The ROIs of **(B)** intratumor(T), **(C)** peritumoral 0~5mm(P1), **(D)** peritumoral 5~10mm(P2), **(E)** peritumoral 10~15mm(P3), and **(F)** T+P1+P2+P3(CR).

All images were imported into an open-source software ITK-SNAP (Version 3.8.0). The tumor ROI was manually segmented slice-by-slice by an experienced radiologist with over 10 years (DP. Huang), without knowledge of the pathological results. Then, the uAI Research Portal was applied for morphological expansion of intratumor ROI. Previous study showed that it would not reduce the risk of recurrence when the tumor resection margin exceeded 15mm (22). Based on this, we performed peritumor expansion three times, 5mm each time, for a total of 15mm. During the delineation and dilation process of the ROIs, non-pulmonary regions were excluded. The peritumoral area beyond the lung outline was manually erased when the tumor located in paramediastinal, subpleural and other special areas. Therefore, in this study, a total of four ROIs were delineated showed in **Figure 2**, namely T (intratumor), P1 (peritumoral 0-5mm), P2 (peritumoral 5-10mm), and P3 (peritumoral 10-15mm). Subsequently, we established a combined radiomics (CR: T+P1+P2+P3) model by integrating intratumoral and three peritumoral ROI features.

In addition, to evaluate the reproducibility of image segmentation, we randomly selected 20 patients to be re-segmented by Dr. Huang and the other doctor (HP. Zhang, with 1 year of experience in imaging) one month later. Intra-observer and inter-observer reproducibility of radiomics features were assessed using intra- and inter- class correlation coefficient (ICC). A value of ICC ≥ 0.85 was considered indicative of good reproducibility. To eliminate index dimension difference, the extracted radiomics features were standardized into normal distributed z-scores. For feature selection, the least absolute shrinkage and selection operator (LASSO) regression was utilized.

With the selected optimal feature sets, we built prediction models for the MPR of neoadjuvant immunochemotherapy for lung cancer by using five machine learning algorithms, including decision tree, Gaussian process, logistic regression, random forest and support vector machine, and the model with the best predictive capability was reserved for external validation. The performance of the different prediction models in internal dataset was assessed by the cross-validation strategy to protect against overfitting due to the limited amount data. In this study, we used five-fold cross-validation (23): the feature set was split randomly, while, the same ratio of positive and negative patients was kept in each partition. Consequently, training on four-fifths of dataset and validating on the remaining partition in each fold, the process was repeated five times within different subgroups, and thus formed five unlike training/validation sets and obtained an average result.

### 2.6 Statistics

SPSS (version 25.0) and Python (version 3.5.6) were used for statistical analysis. Continuous data was presented as mean ± standard deviation or median (interquartile range), and the differences between groups were compared using independent sample t-tests or non-parametric tests. Categorical data was evaluated using chi-square tests or Fisher’s exact tests to assess intergroup differences. Univariable and multivariable logistic regression were used to identify clinical risk factors with odds ratio (OR) and 95% confidence interval (CI). The performance of the model was evaluated using receiver operating curves (ROC), and the area under the curve (AUC), sensitivity, specificity and accuracy were quantified. The DeLong test was used for the performance comparison between different models. The LASSO was utilized for the radiomics features selection. Calibration curve was applied to determine whether the projected probability matches the actual probability. Decision curve analysis was used to assess the prediction models’ clinical viability. A P-value less than 0.05 (P-value < 0.05) was considered statistically significant.

## 3 Results

### 3.1 Clinical characteristics

A total of 148 patients were enrolled retrospectively, and their baseline clinical characteristics were presented in **Table 2**. The training and internal validation sets consisted of 105 patients from SRRSH, of whom 76 achieved MPR (72.4%). The independent external test set (ZCH) included 43 patients, with 22 achieving MPR (51.2%). The average age of the entire cohort was 63.8 ± 6.3 years, predominantly male (94.6%), and most patients had a history of smoking (59.5%). The majority of patients had squamous cell carcinoma as the histopathological type (77.7%). In both of the two cohorts, there was significant difference in histopathological type between the MPR and non-MPR groups (p < 0.05). In the entire cohort, the majority of patients undergoing neoadjuvant treatment were assessed as stage III lung cancer (77.0%). Moreover, T2 (41.2%) and N2 (58.1%) stage were accounted for the most. The main types of immunotherapy agents employed in the two hospitals include pembrolizumab, tislelizumab, and camrelizumab (31.4%, 23.8%, 19.0% in SRRSH and 23.2%, 37.2%, 34.9% in ZCH, respectively). There was no significant difference in the treatment modality for neoadjuvant therapy between the MPR and non-MPR groups in both cohorts.

**Table 2:** Clinical factors of the entire dataset.

Clinical factor	Entire	Training and internal validation (n=105)			External test (n=43)		
	N=148	MPR (n=76)	Non-MPR (n=29)	*P* value	MPR (n=22)	Non-MPR (n=21)	*P* value
Age	63.8 ± 6.3	64.0 ± 6.3	63.1 ± 6.3	0.5	63.1 ± 6.9	64.7 ± 6.3	0.43
Gender				0.25			0.58
Male	140(94.6)	74(97.4)	26(89.7)		20(90.9)	20(95.2)	
Female	8(5.4)	2(2.6)	3(10.3)		2(9.1)	1(4.8)	
Smoking history				0.60			0.96
Current or before	88(59.5)	35(46.1)	15(51.7)		20(90.9)	18(85.7)	
Never	60(40.5)	41(53.9)	14(48.3)		2(9.1)	3(14.3)	
Histopathological type				0.02*			0.02*
Adenocarcinoma	17(11.5)	4(5.3)	7(24.1)		1(4.5)	5(23.8)	
Squamous	115(77.7)	64(84.2)	20(69.0)		20(90.9)	11(52.4)	
Others	16(10.8)	8(10.5)	2(6.9)		1(4.5)	5(23.8)	
Pretreatment clinical stage				0.74			0.32
II	34(23.0)	18(23.7)	6(20.7)		7(31.8)	3(14.3)	
III	114(77.0)	58(76.3)	23(79.3)		15(68.2)	18(85.7)	
Clinical T stage				0.70			0.46
T1	18(12.2)	9(11.8)	2(6.9)		2(9.1)	5(23.8)	
T2	61(41.2)	28(36.8)	13(44.8)		10(45.5)	10(47.6)	
T3	43(29.1)	24(31.6)	7(24.1)		8(36.4)	4(19.0)	
T4	26(17.6)	15(19.7)	7(24.1)		2(9.1)	2(9.5)	
Clinical N stage				0.41			0.55
N0	18(12.2)	11(14.5)	1(3.4)		4(18.2)	2(9.5)	
N1	34(23.0)	19(25.0)	8(27.6)		4(18.2)	3(14.3)	
N2	86(58.1)	39(51.3)	18(62.1)		13(59.1)	16(76.2)	
N3	10(6.8)	7(9.2)	2(6.9)		1(4.5)	0	
Treatment cycle				0.37			0.13
2	117(79.1)	61(80.3)	22(75.9)		15(68.2)	19(90.5)	
3	25(16.9)	12(15.8)	7(24.1)		4(18.2)	2(9.5)	
4	6(4.1)	3(3.9)	0		3(13.6)	0	
Platinum drugs				0.61			0.37
Carboplatin	82(55.4)	30(39.5)	14(48.3)		18(81.8)	20(95.2)	
Cisplatin	65(43.9)	45(59.2)	15(51.7)		4(18.2)	1(4.8)	
Nedaplatin	1(0.7)	1(1.3)	0		0	0	
ICIs				0.84			0.10
Pembrolizumab	43(29.1)	21(27.6)	12(41.4)		7(31.8)	3(14.3)	
Tislelizumab	41(27.7)	19(25.0)	6(20.7)		10(45.5)	6(28.6)	
Camrelizumab	35(23.6)	15(19.7)	5(17.2)		5(22.7)	10(47.6)	
Sintilimab	16(10.8)	10(13.2)	4(13.8)		0	2(9.5)	
Toripalimab	11(7.4)	9(11.8)	2(6.9)		0	0	
Durvalumab	1(0.7)	1(1.3)	0		0	0	
Penpulimab	1(0.7)	1(1.3)	0		0	0	

Data are presented as mean ± SD. Data in parentheses are percentages. *p<0.05.

MPR, major pathological response; ICIs, immune checkpoint inhibitors.

After performing univariable and multivariable logistic regression analysis, the histopathological type was confirmed as an independent risk factor and then included in the clinical model (p = 0.026; OR = 3.328, 95% CI: 1.155-9.588) (**Table 3**).

**Table 3:** Univariable and multivariable logistic regression analyses of clinical factors.

Clinical factors	Univariable		Multivariable	
	OR (95% CI)	*P* value	OR (95%CI)	*P* value
Age	1.024(0.956-1.098)	0.499		
Gender	4.269(0.675-26.993)	0.123		
Smoking history	0.797(0.338-1.877)	0.603		
Histopathological type	3.328(1.155-9.588)	0.026*	3.328(1.155-9.588)	0.026*
Pretreatment clinical stage	0.841(0.296-2.384)	0.744		
Clinical T stage	0.930(0.587-1.472)	0.756		
Clinical N stage	0.758(0.435-1.321)	0.328		
Treatment cycle	0.981(0.410-2.348)	0.966		
Platinum drugs	0.794(0.349-1.810)	0.583		
ICIs	1.127(0.900-1.411)	0.297		

*p<0.05.

OR, odds ratio; CI, confidence interval; ICIs, immune checkpoint inhibitors.

### 3.2 Selection of the radiomics features

In total, 2264 radiomic features were extracted, including 104 original features grouped as: 18 first-order statistics, 72 texture and 14 shape, and other 2160 features based on images through 25 filters, such as boxmean, wavelet, laplacian, etc. A total of 1,067 features were retained after ICC analysis (**Table 4**). After the feature selection processes as mentioned above, the top features of each radiomics model were selected and presented in **Table 5**.

**Table 4:** Radiomics features distribution (Total and after ICC analysis).

	Features	Total (n=2264)	After ICC analysis (n=1067)
Original	First-order features	18	6
Original	Shape features	14	3
Original	GLCM based features	21	11
Original	GLRLM based features	16	6
Original	GLSZM based features	16	1
Original	GLDM based features	14	5
Original	NGTDM based features	5	2
Filtered	Box mean based features	90	35
Filtered	Additive Gaussian noise based features	90	32
Filtered	Binomial blur image based features	90	32
Filtered	Curvature flow based features	90	30
Filtered	Box sigma image based features	90	64
Filtered	Log based features	360	193
Filtered	Wavelet based features	720	395
Filtered	Normalize based features	90	14
Filtered	Laplacian sharpening based features	90	41
Filtered	Discrete Gaussian based features	90	34
Filtered	Mean based features	90	34
Filtered	Speckle noise based features	90	34
Filtered	Recursive Gaussian based features	90	34
Filtered	Shortnoise based features	90	61

GLCM, gray level co-occurrence matrix; GLRLM, gray level run length matrix; GLSZM, gray level size zone matrix; GLDM, gray level dependence matrix; NGTDM, neighbourhood gray-tone difference matrix.

**Table 5:** The selected radiomics features in different radiomics models.

Radiomics Models	The selected Radiomics Features
T(Intratumor) (n=5)	boxsigmaimage_glrlm_LongRunHighGrayLevelEmphasis wavelet_glcm_wavelet-LHH-Idn wavelet_gldm_wavelet-LHH-SmallDependenceLowGrayLevelEmphasis wavelet_glszm_wavelet-LLL-SizeZoneNonUniformityNormalized wavelet_glcm_wavelet-HHL-Idn
P1(Peritumoral 0-5mm) (n=8)	log_firstorder_log-sigma-2-0-mm-3D-Skewness log_glrlm_log-sigma-4-0-mm-3D-LongRunHighGrayLevelEmphasis wavelet_gldm_wavelet-HHL-SmallDependenceHighGrayLevelEmphasis boxsigmaimage_glszm_SmallAreaLowGrayLevelEmphasis shotnoise_glcm_Imc1 wavelet_glcm_wavelet-LLH-Idn wavelet_gldm_wavelet-LLH-SmallDependenceHighGrayLevelEmphasis mean_firstorder_Maximum
P2(Peritumoral 5-10mm) (n=4)	wavelet_gldm_wavelet-HLH-LargeDependenceEmphasis shotnoise_glcm_Idmn wavelet_glcm_wavelet-LLH-Idmn wavelet_gldm_wavelet-LLL-SmallDependenceLowGrayLevelEmphasis
P3(Peritumoral 10-15mm) (n=3)	laplaciansharpening_gldm_SmallDependenceLowGrayLevelEmphasis shotnoise_ngtdm_Complexity log_glrlm_log-sigma-4-0-mm-3D-LongRunHighGrayLevelEmphasis
CR (Combined radiomics) (n=8)	T_wavelet_glszm_wavelet-LLL-SizeZoneNonUniformityNormalized P1_mean_firstorder_Maximum P1_log_glrlm_log-sigma-4-0-mm-3D-LongRunHighGrayLevelEmphasis P2_wavelet_gldm_wavelet-LLL-SmallDependenceLowGrayLevelEmphasis P2_wavelet_glcm_wavelet-LLH-Idmn P3_shotnoise_ngtdm_Complexity P3_log_glrlm_log-sigma-4-0-mm-3D-LongRunHighGrayLevelEmphasis P3_laplaciansharpening_gldm_SmallDependenceLowGrayLevelEmphasis

The CR model incorporated a total of eight radiomics features as follows: 1) intratumor: glszm_wavelet-LLL-SZNUN; 2) peritumoral 0-5mm: firstorder_Maximum, glrlm_log-sigma-4-0-mm-3D-LRHGLE; 3) peritumoral 5-10mm: gldm_wavelet-LLL-SDLGLE, glcm_wavelet-LLH-Idmn; 4) peritumoral 10-15mm: Complexity, glrlm_log-sigma-4-0-mm-3D-LRHGLE, gldm_SDLGLE.

### 3.3 Development and validation of the prediction models

**Figure 3:**
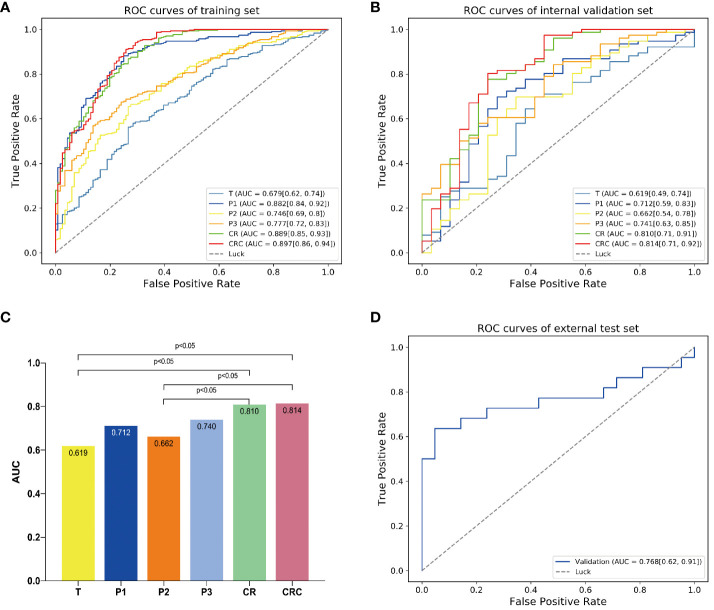
The predictive performance of different models. The AUCs of different models in **(A)** training and **(B)** internal validation sets. **(C)** Delong test showed that the model CR was significantly better than model T. By adding clinical independent risk factor to the model CR, the fusion model (CRC) obtained the best predictive performance [AUC=0.814 (0.71, 0.92)]. **(D)** Receiver operating characteristic (ROC) curve of the fusion model (CRC) in the external test set [AUC=0.768 (0.62, 0.91)].

The predictive performance of each model was shown in **Table 6** and **Figure 3**.

**Table 6:** The performance of different models in training and internal validation sets.

Model	Training set					Internal validation set				
	AUC [95%CI]	Sen	Spe	Pre	Acc	AUC [95%CI]	Sen	Spe	Pre	Acc
Clinical	0.612[0.55, 0.67]	0.947	0.242	0.766	0.752	0.563[0.44, 0.69]	0.947	0.247	0.768	0.752
T	0.679[0.62, 0.74]	0.648	0.604	0.810	0.636	0.619[0.49, 0.74]	0.698	0.587	0.823	0.667
P1	0.882[0.84, 0.92]	0.967	0.347	0.799	0.795	0.712[0.59, 0.83]	0.947	0.200	0.760	0.743
P2	0.746[0.69, 0.80]	0.687	0.655	0.840	0.679	0.662[0.54, 0.78]	0.670	0.660	0.834	0.667
P3	0.777[0.72, 0.83]	0.937	0.293	0.777	0.760	0.741[0.63, 0.85]	0.934	0.273	0.775	0.752
CR	0.889[0.85, 0.93]	0.964	0.613	0.868	0.867	0.810[0.71, 0.91]	0.921	0.533	0.840	0.810
CRC	0.897[0.86, 0.94]	0.977	0.630	0.874	0.881	0.814[0.71, 0.92]	0.947	0.567	0.851	0.838

AUC, area under the curve; CI, confidence interval, Sen Sensitivity; Spe, Specificity, Pre, Precision; Acc, Accuracy; T, intratumor; P1, peritumoral 0-5mm; P2, peritumoral 5-10mm, P3, peritumoral 10-15mm; CR, combined radiomics; CRC, combined radiomics+clinical.

The clinical model showed relatively poor predictive performance in training and internal validation sets (AUC=0.612 and 0.563, respectively). The single ROI radiomics models based on intratumor(T), peritumoral 0-5mm(P1), peritumoral 5-10mm(P2), peritumoral 10-15mm(P3) showed higher AUCs (0.679, 0.882, 0.746, 0.777 and 0.619, 0.712, 0.662, 0.741, respectively) in training and internal validation sets than the clinical model.

The model CR based on Gaussian process demonstrated an AUC of 0.810 for MPR prediction in NSCLC neoadjuvant immunochemotherapy, which is superior than the four single ROI models and significantly improved than the model T (AUC=0.810 vs 0.619, p<0.05). The Delong test showed that the AUC of models CR and CRC was significantly improved compared to models T and P2. However, pairwise comparisons among the remaining models indicated no statistically significant differences in performance (**Figure 3C**). We fused CR model with the clinical model to create combined radiomics + clinical (CRC) model and obtained optimal predictive capability, which achieved an AUC of 0.814, sensitivity of 0.947, specificity of 0.567, precision of 0.851, and accuracy of 0.838 in the internal validation set (**Table 6**).

Finally, the CRC model was validated in an independent external test set and achieved favorable predictive performance, with an AUC of 0.768 (95% CI, 0.62-0.91) (**Figure 3D**).

### 3.4 Calibration curve and decision curve analysis of the prediction models

**Figure 4:**
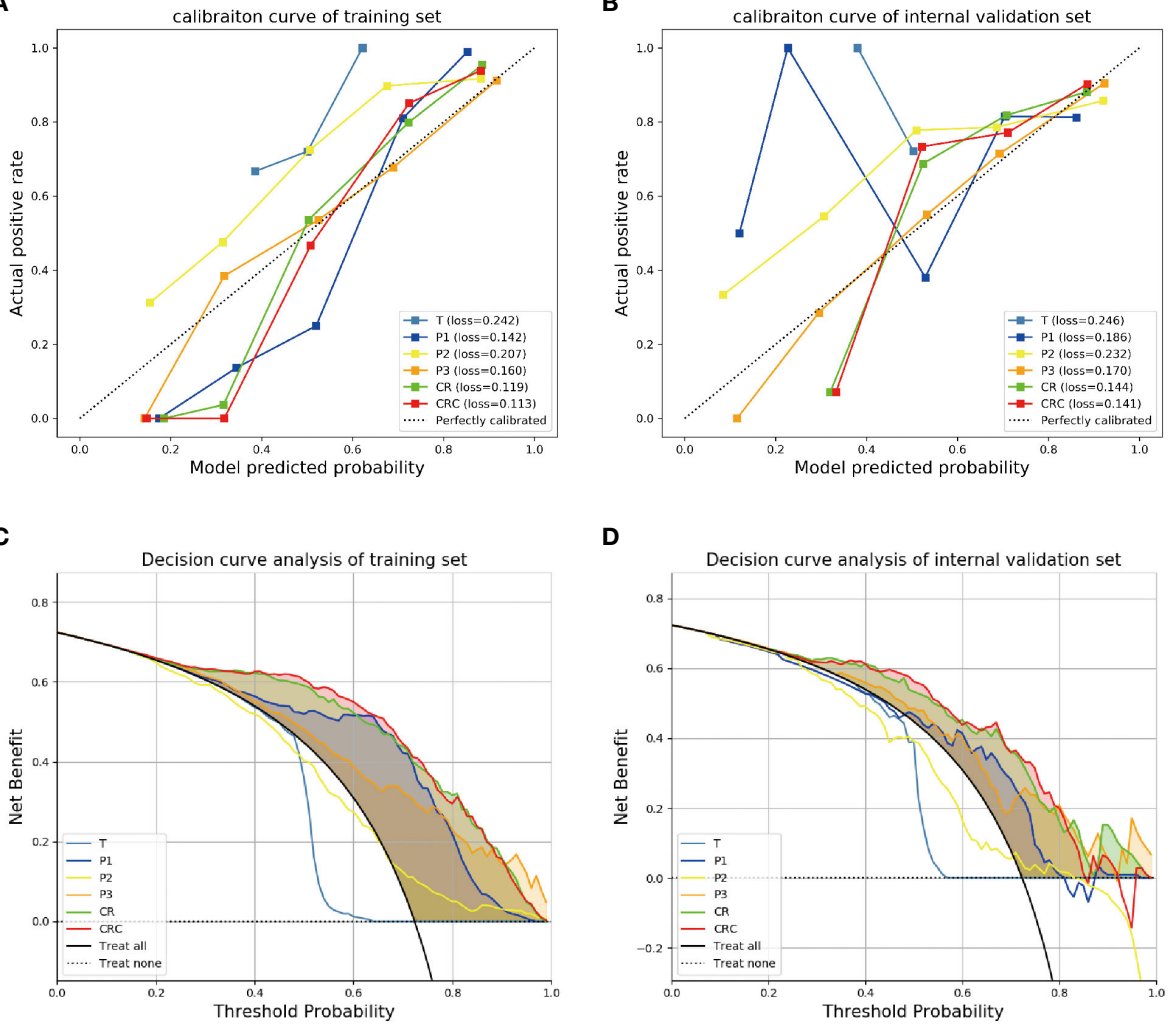
The calibration curves and decision curve analysis for different models. The calibration curves for different models in **(A)** training and **(B)** internal validation sets showed the fusion model CRC had the smallest Brier score loss, which means it has the best predictive performance. The decision curve analysis for the different models in **(C)** training and **(D)** internal validation sets showed that the fusion model CRC provided a better net benefit than other radiomics models for the most of the threshold range.

The calibration curve of the model CR showed that the predicted probability had a good consistency in the internal validation set. And the fusion model CRC had the smallest Brier score loss, which means it has the best predictive performance (**Figures 4A, B**).

Decision curve analysis showed that the fusion model CRC provided a better net benefit than other radiomics models for the most of the threshold range (**Figures 4C, D**).

## 4 Discussion

Neoadjuvant immunochemotherapy has emerged as a promising therapeutic approach for non-small cell lung cancer (NSCLC) (7). However, the evaluation of neoadjuvant treatment efficacy relies on postoperative pathological assessment, leading to time delay. Additionally, the effects of immune checkpoint inhibitors on tumors are complex, and atypical responses such as hyperprogression or pseudoprogression may occur (24, 25), making it challenging to assess the efficacy of neoadjuvant immunochemotherapy through CT follow-up during treatment. Our research showed that the combined radiomics model based on intratumoral and peritumoral regions derived from pre-treatment CT images can predict MPR to neoadjuvant immunochemotherapy in NSCLC. After incorporating the independent risk factor of histopathological type, the model achieved the optimal predictive performance. Furthermore, its predictive efficacy was validated in an external center, indicating its robustness.

Squamous cell carcinoma was identified as an independent clinical risk factor for predicting MPR in neoadjuvant immunochemotherapy in our research, consistent with previous related research (26). A meta-analysis exploring the impact of histopathology on the efficacy of immune checkpoint inhibitors in treating NSCLC showed that immunotherapy can improve overall survival (OS) and progression-free survival (PFS) in both squamous cell carcinoma and non-squamous cell carcinoma, with squamous cell carcinoma patients benefiting more significantly (27). Studies have indicated that compared to non-squamous cell carcinoma, lung squamous cell carcinoma exhibits higher PD-L1 expression, higher tumor mutational burden (TMB), and a greater density of functional TILs in the tumor microenvironment, factors that collectively contribute to the enhanced therapeutic effects of immunotherapy in squamous cell carcinoma patients (28).

In this study, the radiomics model based on intratumoral region had an AUC of only 0.619 (sensitivity of 0.698, specificity of 0.587) in the internal validation group. The peritumoral models showed improvement in AUC compared to the intratumoral model (ranging from 0.662 to 0.741) and higher sensitivity (ranging from 0.670 to 0.947) while their specificity was notably low (P1, P2, P3 = 0.2, 0.273, 0.66, respectively). This pointed out that any radiomics model based on single ROI either intratumoral region or peritumoral region cannot achieve the ideal prediction ability in predicting the effect of neoadjuvant immunochemotherapy in NSCLC.

Prior studies, including one in which our center participated, built prediction models focusing on intratumoral features to predict MPR in NSCLC following neoadjuvant therapy and achieved favorable results (26, 29). Considering that immune checkpoint inhibitors exert their anti-tumor effects by influencing the tumor and its surrounding immune microenvironment (30), it is crucial to investigate the peritumoral microenvironment’s features and their impact on immunotherapy. We extracted radiomics features from both intratumoral and peritumoral regions in arterial-phase, establishing a combined radiomics model with eight top-level radiomics features (one feature from the intratumoral region and the remaining seven from the peritumoral regions). Furthermore, among the seven peritumoral top-level radiomic features, six were texture features, including the common feature (log-glrlm-LRHGLE) from P1 and P3. These results highly suggested that the heterogeneity of the peritumoral microenvironment plays a crucial role in neoadjuvant immunochemotherapy for NSCLC. Moreover, when combining the intratumoral and peritumoral models, there was a significant increase in predicting MPR (AUC=0.810), achieving a relative balance between sensitivity and specificity (0.921 and 0.533, respectively), resulting in a satisfactory accuracy of 0.810.

The tumor microenvironment is composed of fibroblasts, immune and inflammatory cells, as well as interstitial components and microvessels (31). Several studies indicated a correlation between peritumoral texture features and tumor-infiltrating lymphocyte (TIL) density, and higher TIL levels are associated with immune system activation for tumor suppression, indicating a greater likelihood of responding to immunotherapy (32, 33). The distribution of blood vessels in the peritumoral environment also influences the efficacy of chemotherapy and immunotherapy (34). Research by Vaidya P et al. demonstrated that peritumoral texture features can reflect biological pathways such as tumor vascular invasion and neovascularization (35). Disorganized and irregular peritumoral blood vessels promote tumor growth, inhibit the anti-tumor effects of drugs, and are often associated with more heterogeneous radiomic features (17, 35). Additionally, it was demonstrated that different ranges of peritumoral regions are associated with differences in treatment response (36) and exhibit distinct texture feature expressions (37). Our study confirmed a strong correlation between the peritumoral microenvironment and neoadjuvant immunochemotherapy in NSCLC. By incorporating different ranges of peritumoral microenvironment texture features, the prediction model got obvious improvement in predicting MPR.

Although the addition of independent clinical risk factor to model CR resulted in a slight improvement in prediction performance, there was no statistically significant difference in AUC between CR and the best model CRC. This may suggest that information contained within combined intratumoral and peritumoral radiomics adequately capture the efficacy of neoadjuvant immunochemotherapy for NSCLC, thus constraining the representation of clinical factor in the model. However, this requires further verification.

In addition, there is a clear difference in the proportion of MPR patients between the two hospitals included in our study. Indeed, according to a review on neoadjuvant therapy for non-small cell lung cancer, the attainment of MPR varies significantly across different studies, ranging approximately from 36.9% to 84.6% after neoadjuvant immunochemotherapy (10). This variability may be attributed to differences in the patient demographics, disease stages at presentation, and the specific neoadjuvant immunochemotherapy regimens. Based on the aforementioned understanding, we consider the MPR proportions in both hospitals in our study to still fall within a reasonable range. On the other hand, despite the differences in patients and treatment regimens at the two hospitals in our study, our research results still demonstrate that the combined intratumoral and peritumoral radiomics model achieves favorable predictive performance at external center, possibly indicating the effectiveness and robustness of this model.

Our research has several limitations. Firstly, the study was retrospective and might be subject to selection bias. Secondly, while the study included patients receiving neoadjuvant immunochemotherapy, there were variations in the selection of chemotherapy drugs and immune checkpoint inhibitors, as well as differences in the treatment cycles. Therefore, it is essential to unified treatment protocols or conduct a stratified study focusing on different regimens in future research. Thirdly, the imaging data from the two centers were obtained from different manufacturers and multiple models of CT machines, which may introduce inconsistencies in equipment parameters. Lastly, the sample size of this study is limited, and it is necessary to further expand the sample for future research.

In conclusion, our study constructed a CRC model comprising intratumoral and peritumoral features and independent clinical risk factors for predicting MPR in NSCLC patients receiving neoadjuvant immunochemotherapy. The combined model achieved an optimal predictive performance (AUC=0.814), and successfully validated in an external center (AUC=0.768). This provides a non-invasive and effective predictive approach for clinical physicians to identify suitable NSCLC patients for neoadjuvant immunochemotherapy.

## Data availability statement

The original contributions presented in the study are included in the article/supplementary material. Further inquiries can be directed to the corresponding authors.

## Ethics statement

The studies involving humans were approved by Institutional review board of Sir Run Run Shaw Hospital and Zhejiang Cancer Hospital. The studies were conducted in accordance with the local legislation and institutional requirements. The human samples used in this study were acquired from a by- product of routine care or industry. Written informed consent for participation was not required from the participants or the participants’ legal guardians/next of kin in accordance with the national legislation and institutional requirements.

## Author contributions

DH: Writing – review & editing, Writing – original draft, Resources, Methodology, Formal analysis, Data curation, Conceptualization. CL: Writing – original draft, Methodology, Investigation, Data curation, Conceptualization. YJ: Writing – original draft, Methodology, Investigation, Data curation. EX: Writing – original draft, Software, Methodology, Formal analysis, Conceptualization. FX: Writing – original draft, Methodology, Investigation, Data curation, Conceptualization. YG: Writing – original draft, Methodology, Data curation, Conceptualization. RX: Writing – review & editing, Visualization, Methodology, Investigation, Funding acquisition. FW: Writing – original draft, Methodology, Data curation. HZ: Writing – original draft, Methodology, Data curation. KL: Writing – original draft, Investigation, Data curation. LS: Writing – review & editing, Validation, Supervision, Resources, Methodology, Funding acquisition. HH: Writing – review & editing, Visualization, Supervision, Software, Resources, Funding acquisition.
